# Atypical “seizure-like” activity in cortical reverberating networks *in vitro* can be caused by LPS-induced inflammation: a multi-electrode array study from a hundred neurons

**DOI:** 10.3389/fncel.2014.00361

**Published:** 2014-11-03

**Authors:** Francesca Gullo, Alida Amadeo, Giulia Donvito, Marzia Lecchi, Barbara Costa, Andrew Constanti, Enzo Wanke

**Affiliations:** ^1^Department of Biotechnologies and Biosciences, University of Milano-Bicocca, MilanItaly; ^2^Department of Biomolecular Sciences and Biotechnology, University of Milan, MilanItaly; ^3^UCL School of Pharmacy, University of London, LondonUK

**Keywords:** sterile inflammation, LPS, burst activity, neocortical cultures, multi-electrode array, minocycline, TNF-α

## Abstract

We show here that a mild sterile inflammation induced by the endotoxin lipopolysaccharide (LPS), in a neuron/astrocyte/microglial cortical network, modulates neuronal excitability and can initiate long-duration burst events resembling epileptiform seizures, a recognized feature of various central nervous neurodegenerative, neurological and acute systemic diseases associated with neuroinflammation. To study this action, we simultaneously analyzed the reverberating bursting activity of a hundred neurons by using *in vitro* multi-electrode array methods. ∼5 h after LPS application, we observed a net increase in the average number of spikes elicited in engaged cells and within each burst, but no changes neither in spike waveforms nor in burst rate. This effect was characterized by a slow, twofold exponential increase of the burst duration and the appearance of rarely occurring long burst events that were never seen during control recordings. These changes and the time-course of microglia-released proinflammatory cytokine, tumor necrosis factor-alpha (TNF-α), were blocked by pre-treatment with 50 nM minocycline, an established anti-inflammatory agent which was inactive when applied alone. Assay experiments also revealed that application of 60 pM exogenous TNF-α after 12–15 h, produced non-washable changes of neuronal excitability, completely different from those induced by LPS, suggesting that TNF-α release alone was not responsible for our observed findings. Our results indicate that the link between neuroinflammation and hyperexcitability can be unveiled by studying the long-term activity of *in vitro* neuronal/astrocyte/microglial networks.

## INTRODUCTION

Since many years, the term “tripartite synapse” comprising of a presynaptic neuron, a postsynaptic neuron and an astrocyte, was proposed to summarize the evidence from many laboratories, that revealed the existence of bidirectional communication between neurons and astrocytes, especially at level of the synapse (for reviews see Perea et al., 2009). Also, microglial cells [the resident immune cells of the central nervous system (CNS)], play a critical role in neuropathology following brain injury (Perry et al., 2010; Krabbe et al., 2013). In activated microglial cells responding to the injury, migration and phagocytosis are promoted, and inflammatory mediators including nitric oxide (NO), inflammatory cytokines, such as interleukin-1 beta (IL-1β) and tumor necrosis factor-alpha (TNF-α) are released. Since both microglia and astrocytes are present and active in neuronal networks, it is still debated how complicated the cross-talk is between these three cell families (Kettenmann et al., 2013) and interesting mathematical models of this relationship have even been reported in a real-time artificial neuron-astrocyte network (Valenza et al., 2013). It is believed that spontaneous bursting in neuronal network assemblies is physiologically important especially during early development to ensure the reliability of synaptic transmission and information processing (O’Learly et al., 1994; Lisman, 1997; Catalano and Shatz, 1998; Komuro and Rakic, 1998; Garaschuk et al., 2000; Sanchez-Vives and McCormick, 2000; Corlew et al., 2004; Harris, 2005; Dupont et al., 2006).

The aim of the present study was to investigate the proinflammatory effects of low doses of the bacterial endotoxin lipopolysaccharide (LPS) on the spontaneous bursting activity of cortical network cultures derived from *ex vivo* neocortex and containing neurons, astrocytes and microglial cells. Contrary to the *in vivo* network counterpart that is governed by a sensory input and a up- and down-state behavior (see Chen et al., 2013), it is well known that cultured neuronal network activity is reverberating, and long-term data recording can be performed by using *in vitro* multi-site electrophysiology, fully established from many years (Keefer et al., 2001; Beggs and Plenz, 2003, 2004; Gramowski et al., 2004; Tateno et al., 2005; Eytan and Marom, 2006; Gullo et al., 2009, 2010, 2012).

Activity can be basically described by two interlaced time intervals called “burst” (with duration, BD) and “inter-burst interval” (IBI), respectively. The former is characterized by short-lived (i.e., burst-like events, 1–5 s duration, with each neuron having its own duration) and synchronized synaptically mediated neuronal firing from ∼85% of the engaged neurons, and the latter by silent periods of variable duration (7–100 s). Furthermore, by combining electrophysiological multi-electrode array (MEA) recording with fluorescence imaging of γ–aminobutyric acid (GABA)-containing neurons derived from mice expressing green fluorescent protein (GFP), we have previously described how it is possible to classify authentic inhibitory and excitatory cells within the network. Moreover, we showed that the metric which best recognized the firing mode of these different neurons was that defined by the Fano factor (FF; calculated from the ratio between spike-count variance and mean; for details see Becchetti et al., 2012). Thus, we concluded that this statistical analysis demonstrated that BDs of neurons designated as excitatory or inhibitory were qualitatively short or long, respectively.

In each MEA dish, we continuously recorded (over 10s of hours) from a hundred neurons (∼2–3% of network neurons), the BD and excitability changes observed in the two identified clusters of neurons before and after LPS or other pharmacological manipulations. We found that besides a slow increase of excitability of both clusters after LPS, there was a surprising occurrence of atypical burst-like activity, characterized by rare but statistically significant long-duration events, never observed in control recordings before LPS application. Application of low concentrations of the anti-inflammatory agent minocycline (MC) before LPS was sufficient to occlude this hyperactivity. In conclusion, these novel results, obtained from *in vitro* neuronal/astrocyte/microglial networks, reinforce the important, but ultra-slow roles of proinflammatory cytokines (such as TNF-α, whose concentration was measured during LPS experiments) in modulating neuronal excitability as compared to their more well-known faster ionotropic and metabotropic synaptic counterparts. (Santello and Volterra, 2012; Béchade et al., 2013).

## MATERIALS AND METHODS

### CELL CULTURES

Primary cultures of cortical neurons were prepared as previously described (Gullo et al., 2009). Briefly, all of the cerebral cortex (excluding the hippocampus) was removed from decapitated post-natal mice (P1–P3), cut into 1 mm × 1 mm × 1 mm pieces, and digested by trypsin (0.15%) and DNAse (10 μg/ml) at 37°C for 20 min. After enzyme digestion, cells were mechanically dissociated by means of trituration, and plated at densities of 600–900 × 10^3^ cells/ml on glass coverslips (for immunocytochemistry) or MEA Petri dishes pre-coated with polyethyleneimine 0.1% (wt/vol) and laminin 20 μg/ml (30 μm diameter ITO electrodes spaced 200 μm apart, Multichannels System, Germany). Each dish had a recording area of ∼2 mm × ∼2 mm, where on average ∼7000 cells were present but only ∼3000 were neurons. After 3 h incubation, the plating medium was replaced by neurobasal medium (NB) with B27 (Invitrogen, Italy), glutamine 1 mM and basic fibroblast growth factor (bFGF) 10 ng/ml, and the culture was maintained at 37°C in 5% CO_2_. One-half of the medium volume was replaced every 3 days. The cultures in MEA dishes were covered with gas-permeable covers (MEA-MEM, Ala Scientific Instruments, Inc., USA) throughout the culture period [12–22 days-*in vitro* (DIV)].

### CYTOCHEMICAL CHARACTERIZATION OF CORTICAL CELL CULTURES

To characterize the neuronal population in our cultures, we used monoclonal antibodies against microtubule-associated protein 2 (MAP2; 1:1000, Sigma) for neurons, gliofibrillar acid protein (GFAP; 1:500, Dako) for glial cells and the biotin-labeled tomato lectin, biotinylated *Lycopersicum esculentum* agglutinin (b-LEA, 1:500; Vector Inc.) for microglia (Saura, 2007). Controls were performed by omitting primary antibodies or b-LEA. In these cases, no labeling was observed. The cultured cortical cells of control and those treated with LPS (3 μg/ml for 7 h) were fixed at 13 DIV in 4% paraformaldehyde in 0.1M phosphate buffer (PB; pH 7.2) for 20 min, then washed and maintained in PB until cytochemical reactions.

### IMMUNOFLUORESCENCE

After aldehyde quenching with 0.05M NH_4_Cl and permeabilization with 0.1% Triton X-100, cells were preincubated with 1% bovine serum albumin (BSA) for 30 min. Successive incubation was performed for 48 h at 4°C in a mixture of anti-MAP2 and anti-GFAP primary antibodies and b-LEA in PBS/BSA 0.1%. This procedure was followed by incubation with a solution of PBS/BSA 0.1% containing the secondary antibodies DAM-Cy3 (polyclonal, anti-mouse IgG, made in donkey; 1:200 dilution; Cy3 fluorochrome; Jackson Immunoresearch Laboratories) and DAR-Cy5 (polyclonal, donkey anti-rabbit IgG conjugated to the indocarbocyanine Cy5, 1:200, Jackson Immunoresearch Laboratories) to reveal monoclonal and polyclonal antibodies, and Alexa-488-labeled streptavidin (1:200, Molecular Probes) for b-LEA lectin cytochemistry. After rinsing, samples were mounted on coverslips with PBS/glycerol and inspected with a TCS-NT (Leica Laserteknik GmbH) laser scanning confocal microscope, to visualize triple fluorescent labeling. The original emission color of fluorochrome conjugated to secondary antibody DAR-Cy5 has been set to blue to facilitate visual inspection of confocal images. All the thickness of cell cultures (about 12 μm) was acquired by digital superimposing of at least 10 serial optical sections.

### LEA PEROXIDASE CYTOCHEMISTRY FOR MICROGLIAL CELLS COUNTING

Fixed cultures were pre-treated as previously reported for immunofluorescence, except for the additional H_2_O_2_ treatment (0.1% for 5 min) to block endogenous peroxidases. Overnight incubation at room temperature in a solution containing b-LEA in PBS/BSA 0.1% was followed by treatment with the avidin-biotinylated complex (ABC kit, Vector Inc., diluted 1:100) for 75 min and then with a freshly prepared solution (0.075%) of 3-3′-diaminobenzidine tetrahydrochloride (Sigma) and 0.002% H_2_O_2._ Finally, coverslips were dehydrated, cleared and mounted over clean slides. We selected four different cultures/coverslips per experimental set (controls/LPS-treated) from two animals. b-LEA positive (+) cells were counted in four non-overlapping random fields of the coverslips (340 × 250 μm). Image J software was used to count cells on acquired images. Statistical comparison was made using Student’s *t*-test with *P* < 0.05 accepted as significantly different from control.

### TNF-α CONCENTRATION MEASUREMENTS

In each MEA dish, small (150 μl) aliquots of the incubation medium were collected in control and during different times (30 min, 3, 6, 12, and 24 h) after the addition of LPS (3 μg/ml) or MC (50 nM) + LPS. Samples (50 μl) were analyzed in triplicate for murine TNFα with enzyme-linked immunosorbent assay (ELISA) kits (KCM3012, Invitrogen, Italy) accordingly to manufacture’s instructions. The data were expressed as pg/ml following interpolation on the basis of a standard curve. As suggested by the manufacturer, the inter-assay and intra-assay variability were 3.5–4.3 and 2.6–8.2%, with a lower limit of detection <3 pg/ml. During the analysis we did not correct the data for the small change in the total incubation medium volume resulting from the aliquots removal. Data were analyzed using GraphPad 4.0 software employing Student *t*-test for group comparison. *P* < 0.05 was considered statistically significant.

### MEA ELECTROPHYSIOLOGY

#### Drug application: general aspects

As previously described (Gullo et al., 2009), we report results obtained within a few hours after the MEA dish positioning into the incubator, which can thus be considered at the steady-state. The recording area in our MEA dishes was ∼2 mm × ∼2 mm, and in this area, the average number of neurons was in the order of ∼5000, plus about the same number of astrocytes (see Figure 1 of supplementary material in Gullo et al., 2010 at http://journal.frontiersin.org/journal/10.3389/fncir.2010.00011/full) and the average space between cells was therefore relatively large. The drugs were kept as frozen stock solutions in distilled water (or DMSO < 0.1%) at -20°C, until diluted as appropriate with MEA culture medium before each experiment. All experiments were performed by adding the drugs in volumes that were always <1% of the total conditioned media volume bathing the neurons. When indicated, a washout was carried out with a solution pre-conditioned by the same network under control conditions. Murine TNF-α (T7539), thalidomide (THAL) and MC were purchased from Sigma, Italy. PPADS was kindly received from Prof. P. Illes, Rudolf-Boehm-Institut, Leipzig, Germany.

#### Recordings, waveform acquisition, and sorting

We used the same procedures previously described in Gullo et al. (2009, 2010). Briefly, analog signals sampled at 40 kHz were recorded at 36°C in CO_2_-controlled incubators using MEA-1060BC or 1060INV pre-amplifiers (bandwidth 1–8000 Hz, Multichannel Systems, Germany) connected to a MEA Workstation (bandwidth 100–8000 Hz, Plexon Inc., USA). Data were sorted into timestamp files by the MEAWorkstation Sorter software (MEAWS, see details below) and cleaned of artifacts using the OFFLine Sorter program (Plexon Inc., USA). Next, during the PCA-based waveform sorting and for multi-unit electrodes, we applied one of the following procedures: (i) spike removal with a Mahalanobis threshold in the range 1.8–1.4; we concurrently checked that the *P-*value of multivariate ANOVA sorting quality statistics was <0.01 amongst the identified units; (ii) when the previous procedure led to excessive spike invalidation, we manually removed the spikes invading the adjacent unit ellipsoids (the latter method was very effective in decreasing the *P*-values, with only a limited number of erased spikes).

#### Neuronal cluster classification

The method of neuronal classification into excitatory or inhibitory is described in detail in Gullo et al. (2009, 2010) and Becchetti et al. (2012). For each identified unit and each burst, the following characteristics were computed in defined time segments: the autocorrelation function (ACF), the BD (see for recent details in Becchetti et al., 2012), the spike number (SN), the spike rate (SR), the intra-burst SR (IBSR), the IBIs and the FF (Baddeley et al., 1997). We classified the neurons on the basis of an unsupervised learning approach consisting of data reducing principal component analysis (PCA) based on FF as a feature (Becchetti et al., 2012), followed by the K-means clustering procedure. The large differences in these burst metrics was the basis for adopting FF as the best feature to clusterize neurons. As previously described (Becchetti et al., 2012), these procedures normally separated two statistically different clusters composed of numbers of excitatory (∼60–90) and inhibitory (∼15–25) neurons whose ratio always fitted the ratio present in the neocortex, i.e., from 4 to 5 (Gullo et al., 2010; Sahara et al., 2012).

#### Advanced burst classification into states and BD histograms

The global network burst structure was analyzed with standard techniques (see Ham et al., 2008) as well as procedures recently developed by us (Gullo et al., 2012). Briefly, we applied a running window of variable duration (10 ms to 1 s) in order to search for the start and end of the burst duration and collect all of the spikes which were precisely tagged to the engaged excitatory and inhibitory neurons already designated as previously explained above. In conclusion, for each spike, we knew exactly which neuron fired it, in which specific burst it was and how many other spikes were fired. This had the consequence that we could also compute for this neuron the average propensity for firing, namely, its “excitability” in terms of spikes per burst. Our new procedure consisted of performing a classification of all bursts into states (for simplicity here, we chose four), controlled by a PCA based on both BD and SN features. Accordingly, for each of these four states, we could obtain not only the total amounts of SN and the engaged neurons, but also their average time histogram spike number time histograms (SNTH) which specifically described the average time course of each burst from its start to its end (separately for excitatory and inhibitory neurons). Moreover, we computed also the cumulative distributions of BD (BD cum. probability). These last data were found not to be normally distributed (*P*-value < 0.0001, Shapiro-Wilk, Anderson-Darling, and Lilliefors tests by XLSTAT option Normality test, not shown).

#### Data analysis and statistical significance

All of the data are expressed as mean values ± SEM, with *n* indicating the number of experiments. For the non-normally distributed data, we used the non-parametric tests available from XLSTAT-Pro software (Addinsoft, USA). To compute statistical significance among BD cumulative distributions originating from different experiments in general, we preferred the non-parametric equivalent of the ANOVA (a test valid only for normally distributed data), i.e., the Kruskal-Wallis test, where the optional “multiple comparisons” method allowed us to identify various putative groups (we tested the Dunn and the Steel-Dwass-Critchlow-Fligner methods, with the Bonferroni correction). The data were analyzed and the figures prepared using OriginPro 7.0 software.

## RESULTS

### THE ACTIVITY PATTERN IN CONTROL (UNTREATED) AND IN LPS-TREATED NETWORKS

**FIGURE 1:**
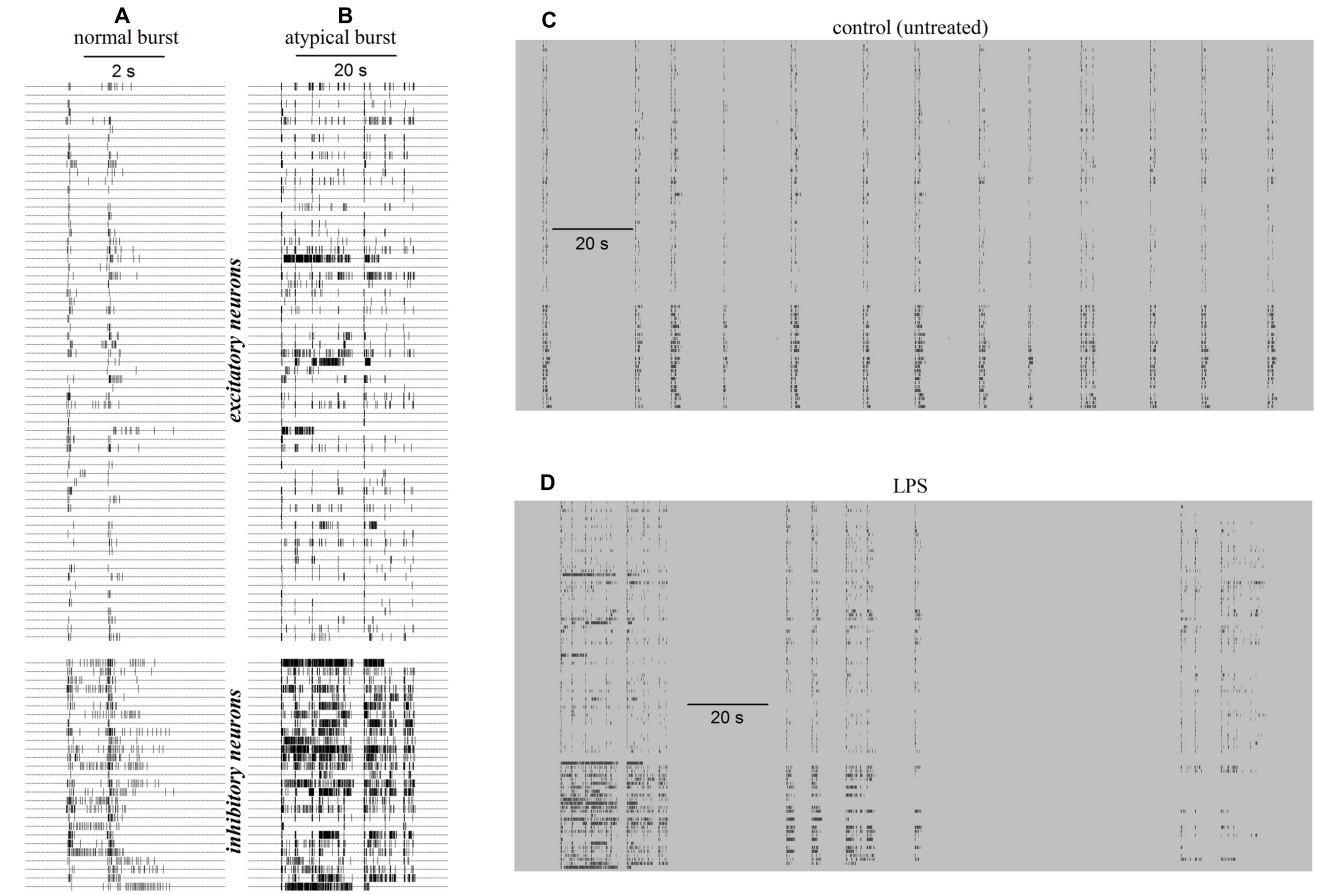
**Raster plots of exemplary normal and atypical reverberating bursts of the network activity recorded in a neocortical/astrocyte/microglial co-culture grown on an multi-electrode array (MEA) dish in control (CON) and 6 h after application of 3 μg/ml lipopolysaccharide (LPS).** Data in **(A–D)** were recorded in control and in LPS, respectively. Small vertical ticks on horizontal lines are timestamps of spikes elicited by identified neurons as indicated. **(A)** Raster plot of a normal burst recorded in control before adding LPS. Note the time scale bar of 2 s. **(B)** Raster plot of an atypical burst recorded 6 h after adding LPS. Note the time scale bar of 20 s. **(C)** Plot of 13 similar normal bursts recorded in control during a time window of 200 s. **(D)** Plot of two atypical bursts recorded in LPS during 200 s (the first is shown in **Figure 1B**). The timestamps in **(C,D)** had the same pattern as in **(A,B)**, i.e., upper and lower ticks relate to identified excitatory and inhibitory cells, respectively.

The simplest way to observe the reverberating burst activity of long-term cultured networks is to examine raster plots from multisite recordings; these show the temporal sequence of action potential timestamps recorded simultaneously from ∼2% of the total number of neurons attached on a square area of 2 mm × 2 mm forming the recorded network. Typical raster plots of a reverberating burst in control during 5 s of activity is shown in **Figure 1A**, where each line corresponds to one cell (the small vertical ticks are the spike timestamps) and the 65 upper and 27 lower lines belong to units classified as excitatory and inhibitory neurons respectively (Becchetti et al., 2012). **Figure 1B** shows the very different properties of an atypical burst recorded 6 h after the application of 3 μg/ml LPS in the same MEA dish (notice the change by a factor of ∼6 on the time scale). While the burst duration (BD) in control (untreated) was around ∼2–3 s, the application of LPS introduced a dramatic BD increase. Similar data were observed in 9 out of 10 different experiments. It can be seen that in 200 s windows, as shown in **Figure 1C**, in control before LPS, the duration of the spontaneous bursts (i.e., thickness of the vertical columns) was characterized by a distribution shifted toward short BDs. In contrast, in the presence of LPS (**Figure 1D**), the appearance of long atypical BD events increased significantly, suggesting an overall increase in neuronal excitability.

**FIGURE 2:**
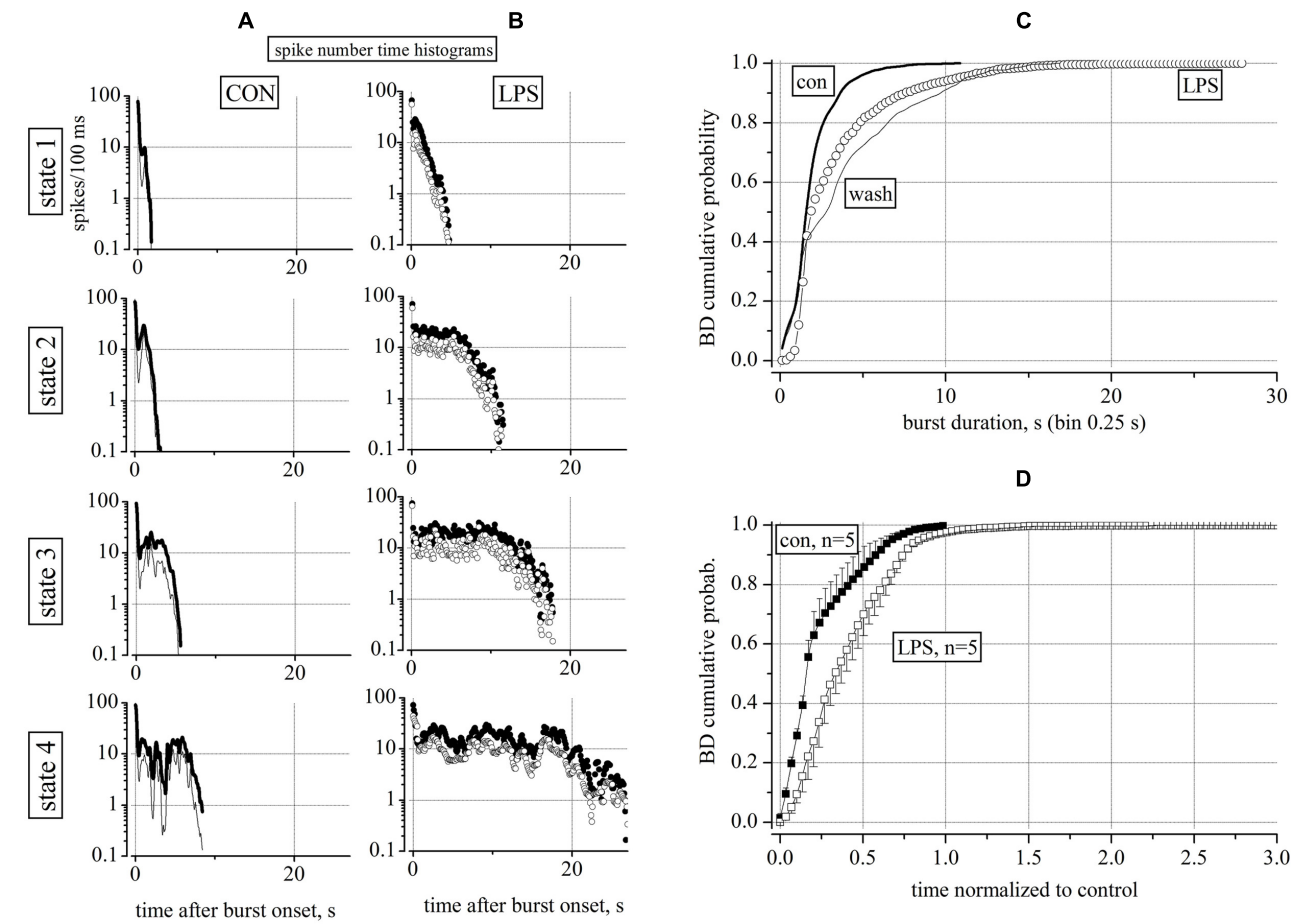
**Characterization of reverberating activity recorded in control and 4 h after application of 3 μg/ml LPS.** Data (time segments of 2 h) in **(A,B)** show global firing properties during control (CON) and 4 h after application of LPS. **(A,B)** Plots of spike number time histograms (SNTH, spike number/0.1 s bin width: log-scale vs. time after burst onset in seconds) representing properties of bursts classified into four states from up to down. In control and in LPS (in parentheses) the number of events assigned to states 1, 2, 3, and 4 were: 122 (280), 255 (50), 66 (20), and 15 (6). LPS therefore strongly modified the pattern of the burst durations by favoring the large (>10 s) durations in 21% of the bursts as compared to control where all of the durations were below 10 s as shown in detail below in **Figure 3**. **(C)** Superimposed BD cumulative probability plots during control (5 h, thick line), 3 μg/ml LPS (6 h, open circles) and after washout (4 h, thin line). Data from an experiment with 65 excitatory and 26 inhibitory neurons (classified as described in Section “Materials and Methods”) respectively. Number of analyzed bursts were 1208 in control, 1421 in LPS and 634 in washout respectively. The non-parametric Kruskal–Wallis statistical significance method gave, for the pairs con/LPS, LPS/wash and con/wash, the following results: *P* < 0.0001, *P* < 0.029, *P* < 0.0001, respectively. **(D)** Cumulative probability of BD averaged from 5 similar experiments done in different dishes after correctly normalizing the control part of each experiment for having a value of 1 and 60 equally spaced bins (error bars are ±SEM). Note that the LPS action on BD distribution was consistently different with respect to control; also for LPS data, after normalization, the time scale had a maximum value three times longer than for control data.

To investigate the underlying properties of this activity, we performed a statistical analysis of the neuron type (excitatory or inhibitory, see Becchetti et al., 2012) and a classification of the durations of the acquired bursts (see Gullo et al., 2012). We found that, on average, the number of engaged neurons was not changing before and after LPS (not shown), but on the contrary, it was the number of elicited spikes (SN, in each burst) that was strongly increasing for both neuronal types (clusters). To quantify the heterogeneity of the BDs, we performed a classification procedure of all bursts into four states according to two features, namely, SN and BD. The assignment of each burst to one of the four states was performed automatically, and the states were significantly different according to our classification procedure (see Materials and Methods). During a typical whole experiment of 14 h, we computed the data in sequential time segments of 2 h in control (6 h) and after the LPS application (8 h). Each burst was characterized by how many spikes were elicited from burst onset up to its end; we then computed the temporal histogram of the SN (SNTH: Gullo et al., 2010, 2012) and plotted it by averaging all data in each 2 h time segment and among all the neurons belonging to the same cluster (see Materials and Methods). The SNTH data plots observed in control (untreated) and in LPS are shown in **Figures 2A,B**, respectively: for excitatory cluster: thin lines and open circles and for inhibitory cluster: thick lines and closed circles. During control, state 1 and 2 maximal BDs where in the range 2–3 s and for states 3 and 4 BDs were up to 6–7 s. In contrast, in LPS, the BDs were much longer: for states 1 and 2 BDs reached the range 5–11 s, but for states 3 and 4 they were the longest from 18 up to 27 s (for details see Figure legend).

Taken together, these results suggest that LPS was able to barely modify the short burst states, but, in contrast, it largely increased the number of spikes of the long-lived atypical bursts compared to the control recording condition. This could indicate that the network (balanced in control and with burst ending on average after 6–7 s), was shifted toward a new steady-state characterized, in both neuronal clusters, by an increased excitability which favored a longer and atypical activity in bursts.

Although the SNTHs shown in **Figures 2A,B** clarified the temporal structure of the bursts in control conditions and in LPS, it was important to check that these results were complemented by an analysis independent from the burst classification method. To this aim, we used a standard cumulative probability analysis of BD as shown in **Figure 2C** for an exemplary experiment in which we could obtain also a tentative washout from LPS. These results were useful to better demonstrate the crucial effects of LPS: (1) in control at relatively long BDs, i.e., ∼7 s, ∼99% of bursts had BDs smaller than 7 s, (2) but in LPS, only 95% of the bursts had BDs <7 s and the rest of the bursts had longer BDs; (3) in LPS the short (<1 s) BD bursts practically disappeared, but, (4) on washout, the short bursts reappeared but the long ones did not disappeared, suggesting a non-completely washable effect of LPS. Since both the distribution of BD and its cumulative probability were not normal, a non-parametric Kruskal–Wallis statistical analysis (see Materials and Methods) was performed here and will be used in all of the following results. As indicated in the legend of **Figure 2**, this analysis suggests that the LPS data were highly significantly different (*P* < 0.0001) from both control untreated and washout, but control was different from washout with a *P* < 0.029, indicating a lingering action of LPS.

Since in each MEA dish, it was practically impossible to exactly control the precise composition and the amount of cells at div 0, each network started its activity and became mature at slightly different days. This implies also that each dish had different intrinsic properties such as the average values of BD, the IBIs, the total number of neurons and the ratio between the excitatory and inhibitory cells. These and other variables such as the density of astrocytes and microglia normally could in principle, produce different cumulative histograms. To average different experiments it was necessary to normalize the time scales of each LPS experiment to its control and then average data. From 5 independent and successful experiments, we plotted in **Figure 2D** the averaged control data (*n* = 5) of the control time segments (closed squares) and LPS segments (open squares) which proved to be significantly different (*P* < 0.0001). The averaged control “con” data had *P* > 0.05 with respect to each single control experiment, thus suggesting that they were not statistically different after normalization. The same was true for the LPS data.

On the whole, these results point out that the robust modifications in firing activity induced by LPS were long-lasting at least for 4–6 h during washout, although we observed only a weak recovery of short bursts. The data points of the LPS trace illustrate that novel bursts were also present in a BD range in which it was always impossible to capture such bursts during control. On the contrary, in the range from 0 to ∼2 s the histograms were hardly different from each others.

### LPS DID NOT AFFECT ACTION POTENTIAL WAVEFORM NEITHER IN EXCITATORY NOR IN INHIBITORY NEURONS

**FIGURE 3:**
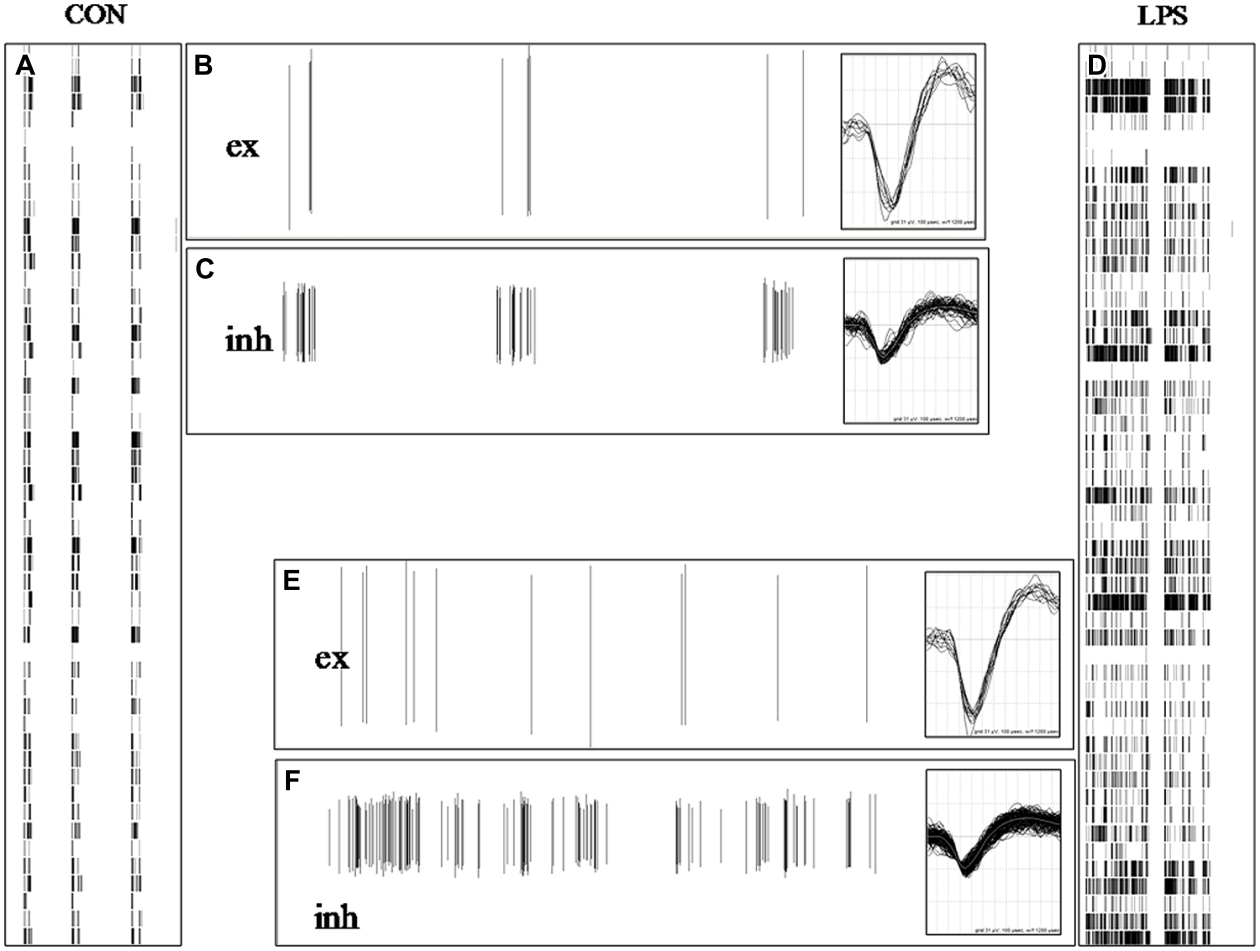
**Lipopolysaccharide did not affect action potential waveform neither in excitatory nor in inhibitory neurons. (A,D)** 35 s-long windows in which are shown raster plots of timestamps of normal and atypical burst in control (CON) and 6 h after adding LPS (each point in a column contains data from one electrode). In **(A)** are present three brief bursts and in **(B)** one long burst in LPS (identified as one single up-state because the short; <3 s) inter-spike interval was well below the average IBI value of ∼15 s. **(B,C,E,F)** 35 s-long windows showing plots of spike amplitude (vertical lines) vs. time in control and during LPS for identified excitatory **(B,E)** and inhibitory **(C,F)** neurons in two different electrodes. The number of identified excitatory and inhibitory cells was 69 and 24, respectively. The insets associated with **(B,C,E,F)** show the superimposed spikes recorded in the respective windows and their vertical and horizontal dimensions correspond to +62/-93 μV and 1.2 ms, respectively.

The results shown in **Figures 2A,B** suggested that the statistical analysis of the SNTHs in control and in LPS at a concentration of 3 μg/ml, differed dramatically for both neuronal clusters. The diversity was present not only in the BD that was much longer in LPS, but also in the mean SN of the inhibitory clusters, with respect to the excitatory clusters (values of closed symbols were always greater than those of open symbols). This consistent observation suggested to us to investigate in detail both the timing pattern of bursts and the waveforms of the action potentials in both types of clusters. To this aim, we compared the spike timing pattern and their waveforms acquired during control or 8 h after adding LPS, when the effects on excitability had become consistently established. In **Figure 3** we show how spike trains of classified neurons [excitatory (ex) inhibitory (inh)] looked during exemplary events in control and LPS. In a 35 s time-window, the two left (A) and right (D) raster plots (timestamps) show three brief bursts in control and one long burst (separated by a very short interval <3 s) in LPS, respectively. From two different electrodes, we identified two neurons classified as belonging to the excitatory (B,E) and inhibitory (C,F) clusters and we show how their spike trains (vertical lines) contributed differently to the global network burst. In the control windows, B and C, the spikes were rare or abundant according to the different modes of firing of excitatory and inhibitory cells, and the same pattern can also be followed in the companion windows E and F recorded in LPS. To investigate if the spike waveforms were changing from control to LPS, we superimposed the action potential waveforms relative to these bursts (see the insets in B,C and E,F); it is clearly evident that on average, they did not differ. Since no detectable changes were also observed at the level of IBI (the average time between bursts), a variable that is linked to the average probability of starting a burst, it is reasonable to assume that the average resting membrane potential of the engaged neurons was not dramatically affected by LPS, despite their increased propensity toward burst broadening. This observation suggests that the LPS-induced actions on excitability described above were quite subtle, perhaps originating from time-dependent alterations in the properties of voltage-gated ion channels responsible for maintaining the neuronal excitability (see Due et al., 2012); however, this would need to be confirmed in intracellular recording experiments from individual neurons.

### THE TIME COURSE OF LPS ACTION

To further investigate the time-course of development of the LPS effects illustrated in **Figures 1** and **2** we re-analyzed some experiments by dividing the control and the LPS periods into shorter half-hour segments in order to check if during briefer periods of time, there were relevant fluctuations that were averaged out when longer segments were used. At the same time, a half-hour sampling had the advantage of verifying the exact time course of the changes introduced by LPS. This opened the possibility that some of the long bursts observed in control could be confounded or blurred with those observed during the LPS application. Furthermore, we wanted also to verify if briefer segments of control data could be considered statistically indistinguishable among themselves, and also to verify that the same concept was valid for the LPS-data when the action of the drug reached its final maximum steady-state level.

**FIGURE 4:**
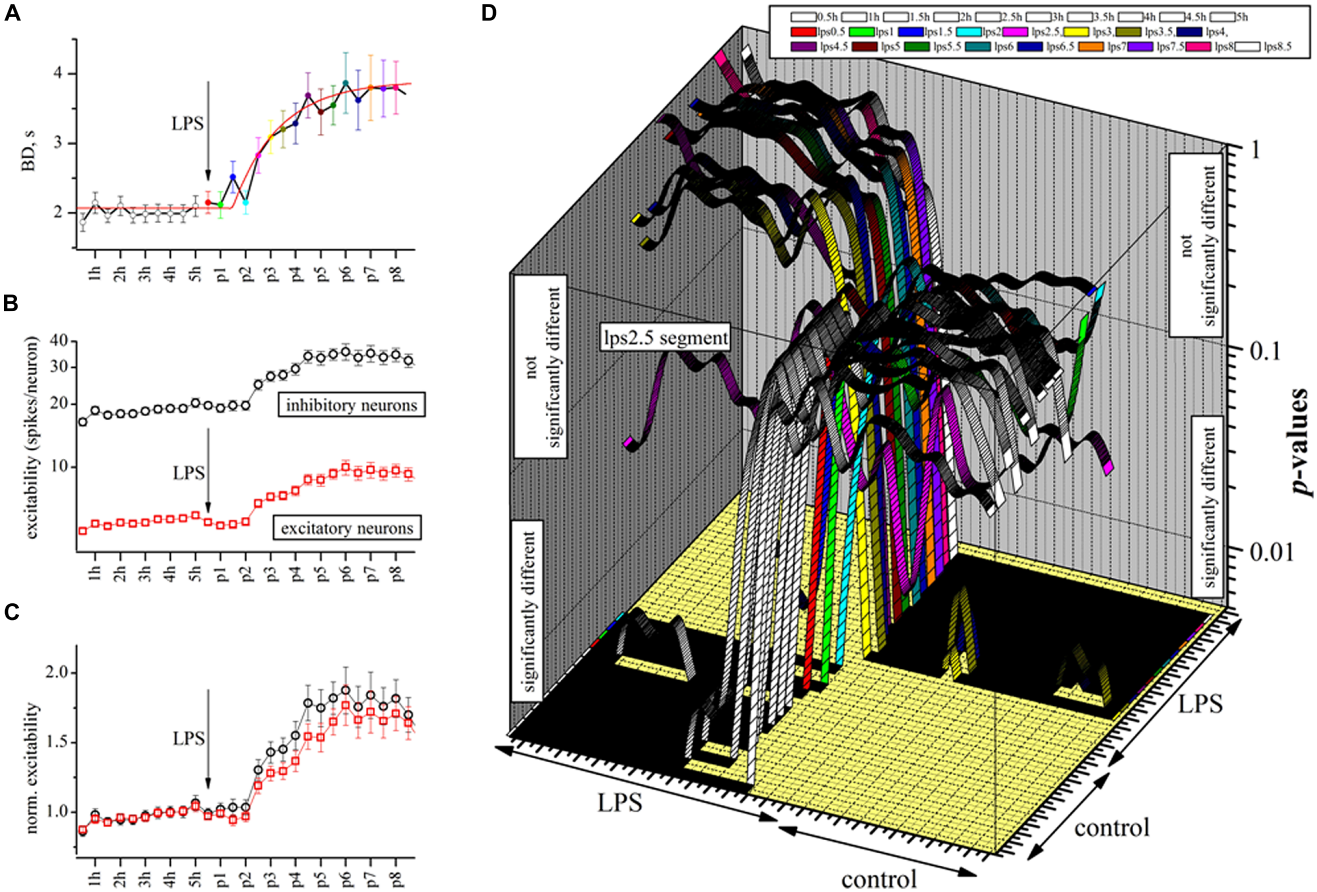
**The delayed excitatory action of LPS studied at a half-hour resolution by analyzing BD and engaged excitatory and inhibitory neuron excitability. (A)** Plot of BD in an exemplary experiment where time was divided into half-hour segments (10 in control and 16 during LPS). The red line is the best fit to an exponential function of the following type: fit line = 2.07 + 1.9*[1 - exp[-(x - τ_d_)/τ_LPS_]], where τ_d_ and τ_LPS_ are in seconds and represent the delay (5250 ± 970 s, *n* = 13) and the LPS onset time constant (8355 ± 2980 s, *n* = 13) which characterized the drug action, respectively. **(B)** For the same experiment, plot of excitability, defined as the average number of spikes elicited by engaged neurons in the same cluster in each up-state (red and black for excitatory and inhibitory cells). **(C)** For the same experiment, the data shown in **(C)** but normalized to the mean control data for each cluster (black and red symbols for inhibitory and excitatory neurons, respectively). Note the slow time course of excitability increase was similar in both neuron types. **(D)** 3D graph where each ribbon is the *P*-value of significance plotted vs. 27 time segments in the exemplary experiment of **(A–C)**. There are 27 such ribbons plotted in order to evaluate all of the 702 (but those significant were 351) multiple pairwise (those identical were omitted) comparisons to compute the statistical significance by the non-parametric Kruskal–Wallis test (Dunn analysis, Bonferroni correction). Each ribbon is for each 0.5 h segment (open ribbon for control, colored ribbons for LPS data). Control (before) and LPS regions (succeeding) are indicated. Since the vertical axis of the *P*-values is in the form of a log-scale whose middle point is 0.05, all the regions where ribbons are above or below 0.05 represent non significant or significant comparisons, respectively. The time segment “lps2.5” that is in between control and steady-state LPS segments resulted in a “strange” shape. The data show that during control and well after LPS application, the BD data are significantly similar; however, during the development of the LPS action, they slowly (after each half hour) changed consistently to the differences shown in **Figures 3A–C**.

The BD data are shown as points in the time plot of **Figure 4A** where the tentative fitting to an analytical function describing drug onset time-course is shown by the red superimposed line. We found that the fitting of these data to an exponential relationship allowed us to suggest that the process really consists of a delayed exponential, namely *y* = exp - (t - τ_0_)/τ_LPS_, where τ_0_ and τ_LPS_ are the delay and LPS action time constants found to be ∼1.5 and ∼2.3 h, respectively. In conclusion, and taking into account the other successful experiments, we found that LPS, at a concentration of 3 μg/ml, was able to produce a considerable and significant increase in the BD duration in the neuronal cultures.

Given the fact that we never observed any significant change in the spike waveform in the presence of LPS (see **Figure 3**), we wanted to examine how these effects on BD could be interpreted at the level of neuronal excitability. Since in our experiments, we continuously sampled the same set of neurons, we could count the number of spikes elicited by each engaged cell during each burst. This analysis is shown at the same half-hour resolution in **Figure 4B** for the two clusters of neurons. The plot illustrates that excitatory and inhibitory cells behaved in a very different manner, the former class being much less active than the latter class, possibly indicating that they were under a strong “braking” influence from local inhibitory neurons as already shown in Gullo et al., 2010). A further interesting analysis is shown in **Figure 4C** where we normalized the previous data by the control values (averaged) to remove the different firing modes and this showed that despite their different background levels of activity, both clusters behaved similarly in the presence of LPS (*P* > 0.05, *n* = 150). We did not observe any other change in the activity properties like IBI, except for an obvious increase in mean SR of inhibitory and excitatory (in parenthesis) clusters which changed from 1.44 ± 0.08 (*n* = 26) [0.37 ± 0.03, (*n* = 65)] in control to 1.82 ± 0.15 (*n* = 26) [0.48 ± 0.06 (*n* = 65)] in LPS at the experiment end after 7 h. This finding suggests that the LPS action on the neuronal culture resembled a change of the working “set point” of the network activity as previously proposed to explain homeostatic plasticity (for a review see Turrigiano and Nelson, 2004).

To test the random BD changes observed during each half-hour segment, we did the complete study of the statistical significance of the BD data in this exemplary experiment whose behavior is illustrated in **Figures 4A–C** by using the Kruskal–Wallis test (see Materials and Methods). To do so, we computed all of the *P*-values amongst all the sub-segments, both in control and during the action of LPS. In **Figure 4D**, these data are shown in a 3D graph where, on the z-axis, are plotted the *P*-values of the BD statistical analysis originating from the 27 time segments (0.5 h each) of this exemplary experiment (for a total of 351 comparisons, see legend). We found that all the segments in control (10, open symbols) and the first four LPS segments had highly similar properties, i.e., *P*-values >>0.05 before LPS and <<0.05 after LPS, suggesting that the BDs slowly shifted from the properties they had in control to a new stationary and significantly different longer mode. Conversely, when we examined the BD data in the LPS region, the behavior was symmetrical and opposite.

Lipopolysaccharide was not used at higher concentrations because it has been recently shown that at 10 μg/ml, other remarkable effects lead to apoptosis (Nimmervoll et al., 2012). Another 10 experiments performed at lower LPS concentrations of 1 μg/ml produced data that were either transient (3 out of 10) or subject to the intrinsic variability among different dishes (3 out of 10) and therefore were not sufficiently stable and trustable to be analyzed further. At a concentration of 0.3 μg/ml, only one experiment out of 5 was successful (not shown). The slowly developing LPS action described here is completely different from the fast action of any agonist or antagonist of voltage-gated ion channels or excitatory/inhibitory neurotransmitter receptors tested on MEA culture dishes (Gullo et al., 2009, 2010, 2012; Puia et al., 2012).

### LPS EFFECTS CAN BE BLOCKED BY NANOMOLAR CONCENTRATIONS OF MINOCYCLINE

**FIGURE 5:**
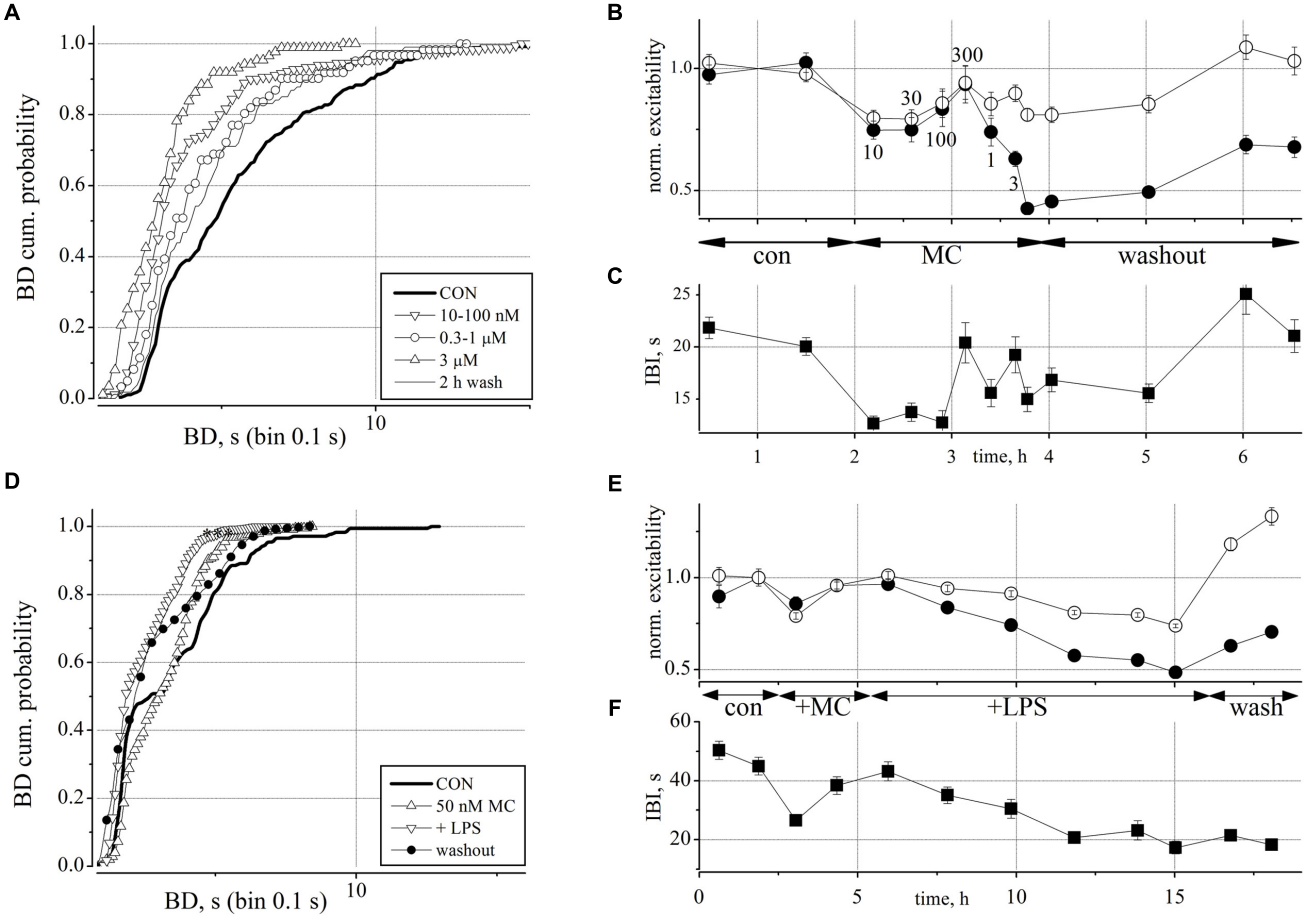
**Minocycline (MC) dose-response relationship and its antagonistic action toward the effects of LPS on neuronal excitability. (A)** Plot of superimposed BD cumulative probability histograms in control (thick line), in 10–30–100 nM MC (inverted triangles), in 0.3–1 μM MC (circles), in 3 μM MC (upward triangles), and after 2 h of washout (thin line). Statistical significance was assessed on the cumulative probability data by Kruskal–Wallis method (*P < 0.05; ***P < 0.0001; ^-^*P* > 0.05) according to the following matrix-table that illustrates in a compact form the 10 *P*-values among all the possible combinations of the different probability curves (see **Table 1**). **(B)** Plot of the normalized excitability of identified excitatory/inhibitory neurons (open/closed symbols). Notice that the effects of the highest MC dose persisted for more than 1 h during washout. The small numbers near symbols indicate the increasing concentrations of MC. **(C)** Plot of IBI vs. time. Notice an early IBI decrease effect at the lowest MC dose. **(D)** Plot of superimposed BD cumulative probability histograms in control (thick line), in 50 nM MC (2 h, upward triangles), after adding 3 μg/ml LPS (10.5 h, inverted triangles), and after 2 h of washout (closed circles). This exemplary representative experiment is similar to other four experiments and shows that only small, i.e., <100 nM doses of MC produce small and transient (<1 h) and recoverable effects on the network activity. Number of analyzed bursts in control, +MC, +LPS and washout were 176, 250, 1300, and 405, respectively. Statistical significance was assessed on the cumulative probability data by Kruskal–Wallis method as follows: CON vs. MC *P* < 0.001; CON vs. LPS *P* < 0.0001; CON vs. WASH *P* < 0.0001; LPS vs. WASH, MC vs. WASH, LPS vs. MC *P* > 0.05. **(E)** Plot of the normalized excitability for identified excitatory (open circles) and inhibitory neurons (closed circles). **(F)** Plot of IBI vs. time.

Minocycline, a semi-synthetic second generation tetracycline analog, has been extensively used therapeutically as an anti-inflammatory agent (for a recent review see Tikka et al., 2001; Filipovic and Zecevic, 2008; Henry et al., 2008; Garrido-Mesa et al., 2013) and also possesses neuroprotective and anticonvulsant properties (Beheshti Nasr et al., 2013; Dodd et al., 2013). Since our LPS experiments had a duration of 8–10 h, we decided to test MC at one of the lowest concentrations used by Tikka et al. (2001) in mixed spinal cord cultures, namely 50 nM. Since preliminary experiments at high concentrations of MC (3 μM) caused a persistent and irreversible strong *decrease* of neuronal activity (not shown), indicating an unwanted possibly toxic action, we wanted to control the MC putative intrinsic activity on our networks by performing some preliminary dose-response experiments as shown in **Figures 5B,C**.

**Table 1:** The 10 P-values mentioned in the Figure 5A legend as Table 1.

	Con	10–100 nM	0.3–1 μM	3 μM
Con				
MC 10–100 nM	*			
MC 0.3–1 μM	*	–		
MC 3 μM	***	***	***	
2 h wash	*	–	–	***

We applied increasing concentrations of MC from 10 nM up to 3 μM to obtain a dose-response curve during short time segments (30, 20, and 10 min). In **Figures 5A–C** we plotted the BD cumulative histograms, the excitability and the IBI data recorded during a 6 h period which started with control (untreated) and finished with the washout. As shown in **Figures 5B,C**, the smallest concentration of 10 nM MC caused both a decrease of excitability and a smaller IBI which, taken together, resulted in a small (but non-significant) *increase* in global spiking rate (SR, not shown) which seems counter-intuitive.

**FIGURE 6:**
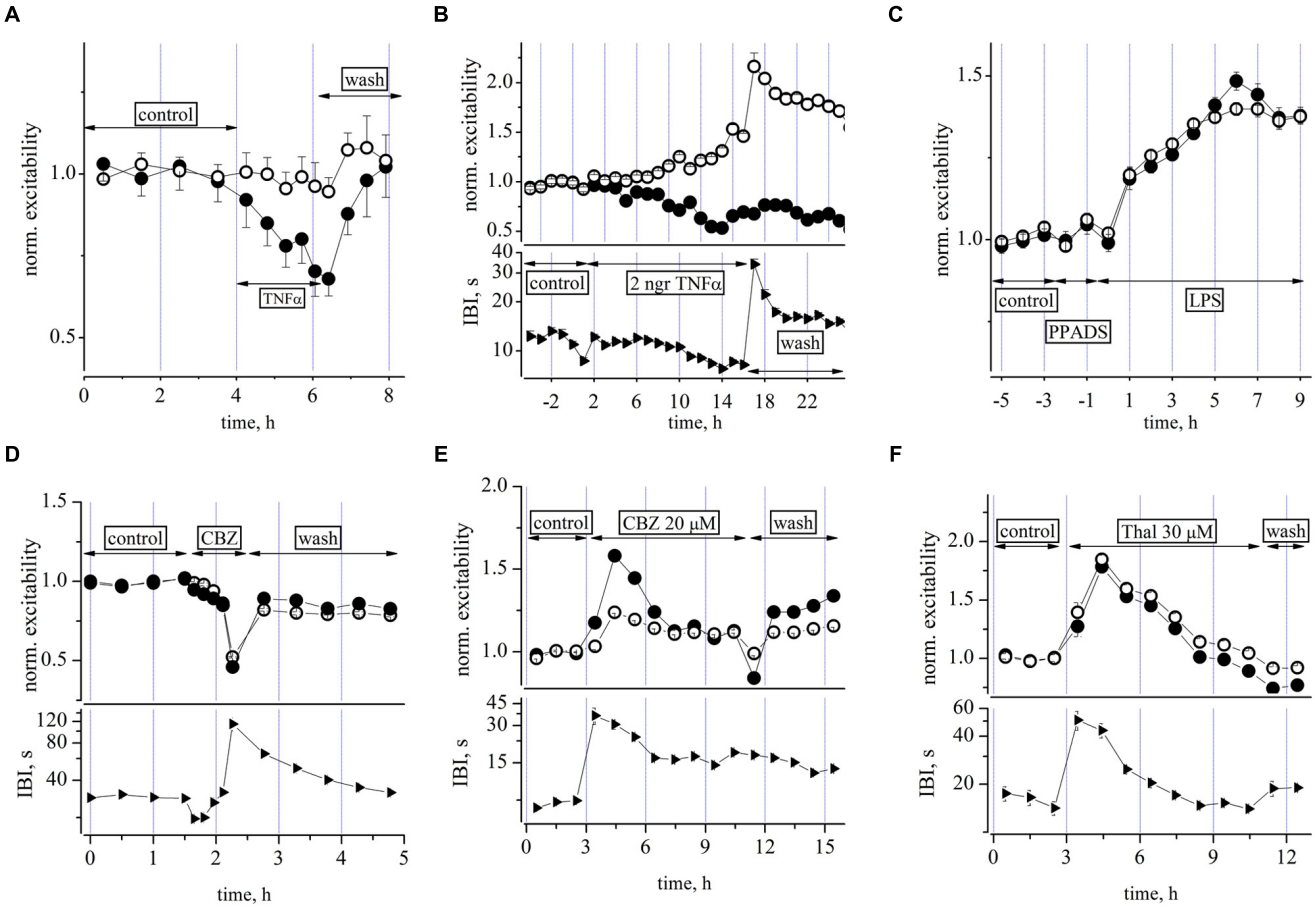
**Effects of TNF-α, PPADS, carbamazepine, and thalidomide on neuronal excitability. (A)** Plot of normalized excitability (relative to control) in an exemplary dose-response 2 h experiment in which increasing concentrations of TNF-α from 10 to 300 pM [see arrows labeled TNF-α, 10 (15 min), 20 (15 min), 60 (25 min), 100 (25 min), 300 pM (15 min)] were applied to a culture while recording from a network of 95 excitatory and 19 inhibitory identified neurons, respectively. No effects were seen on IBI (not shown). **(B)** Plot of normalized excitability (upper) and IBI (lower) vs. time after addition of a single dose of TNF-α see line with arrows and the 60 pM TNF-α label. Notice the slow build-up of an unbalanced neuronal activity characterized by higher and lower excitability in both excitatory and inhibitory neuronal clusters, respectively. According to **(A)** experiment, we expected a small excitability decrease for inhibitory cells, but notice that here the averaging time is longer (3600 s for each symbol) than that of **(A)** (∼900 s). Although it can be seen that ∼3 h after washout, the excitability during the up-states resulted consistently changed, the network SR was not significantly altered. The mean IBI values during TNF-α were not significantly different from control (12.2 ± 0.2 vs. 15.4 ± 1 s, *n* = 21). A network of 70 excitatory and 21 inhibitory identified neurons were recorded, respectively.** (C)** Plot of normalized excitability in control, after the addition of PPADS (50 μM) and the subsequent co-addition of LPS at 3 μg/ml. Network of 86 and 17 excitatory and inhibitory identified neurons, respectively. Note that PPADS did not prevent the excitatory effect of LPS. **(D)** Plot of normalized excitability (upper) and IBI (lower) vs. time for increasing doses of CBZ (1, 3, 10, 30, and 100 μM) and the subsequent washout. Network of 91 excitatory and 37 inhibitory identified neurons, respectively. **(E)** Plot of excitability (upper) and IBI (lower) after the addition of a single dose of CBZ at 20 μM. Notice that the IBI change preceded and outlasted the excitability changes. Network of 99 and 47 excitatory and inhibitory identified neurons, respectively (see explanation in text). **(F)** Plot of excitability (upper) and IBI (lower) after the addition of thalidomide (THAL) at 30 μM. Notice again that the IBI change preceded the excitability change by ∼1 h. Network of 121 and 27 excitatory and inhibitory identified neurons, respectively.

This observation obviously suggests that in our cultured reverberating networks (and putatively in *in vivo* recordings during synchronized spontaneous activity or during sleep, see Discussion) the global activity is controlled by two distinct and functionally independent mechanisms: namely, burst activity (with BD duration) among neurons connected by glutamatergic and GABAergic synapses, with their different properties and therefore subject to different pathways leading to the burst onset and end and, in contrast, the IBIs characterized by quasi-silent periods in which all cells are temporarily hyperpolarized and recovering from the previous excessive firing activity (synaptic fatigue, slow voltage-gated ion channel inactivation, slow metabotropic neurotransmitter action, etc). Indeed, on average, SR (spike/s, a variable computed independently from BD and IBI definition) is proportional to excitability (spikes during the burst), but inversely proportional to IBI (because in IBI there are no spikes elicited). Thus, any excitability change with no IBI change leads obviously to the same SR change. On the contrary, if the excitability decrease is accompanied by a proportionally larger IBI decrease as shown in panel C, the effect on SR can result in a counter-intuitive increase (we show below in **Figures 6D–F** other counter-intuitive results). Since in the two clusters, SRs originate from different excitabilities of different numbers of neurons, we always found that in CNS networks, the average SR of the inhibitory cluster was higher than the SR of the excitatory neuron cluster (Gullo et al., 2010).

This effect persisted up to a concentration of 100 nM but recovered at 300 nM MC where both variables had the values seen in control. Since the successively higher concentrations of MC produced a further decrease in excitability and BD that was difficult to washout (see at the fifth hour), we interpreted the initial, but recoverable effects as a momentary disturbance that the network compensated by its own homeostasis. This was confirmed by the BD cumulative analysis of the data shown in **Figure 5A** (superimposed curves). In the plot, it is possible to follow the data in control (thick line) and the slow left-shift of the curves (symbols) during the increasing MC concentrations (see meaning of different symbols in the legend) and the final washout (thin line) which returned back toward the control curve.

In conclusion, we learned from these experiments that at very low concentrations, the putative early intrinsic action of MC should not interfere with other drugs working at a much slower rate. On the contrary, the specific inhibitory action of MC at higher concentrations on the excitability of inhibitory neurons and not on excitatory cells suggested that at these high concentrations the action was clearly not negligible. The fast but recoverable action of MC we observed will certainly need further investigation in the future.

In order to test whether the excitatory action of LPS could be blocked by MC, we pre-incubated the networks with 50 nM MC for 2 h, and then added LPS, in order to exclude the transient effects of MC mentioned above. The experiments were analyzed similarly to those shown in **Figures 2** and 3 and one exemplary test (out of 5) is illustrated in **Figures 5D–F**. We found that after pre-treatment with MC, LPS no longer produced rightward shifts of the BD probability to indicate significant BD increases (*n* = 5; cf. **Figures 2C,D**). This is shown in **Figure 5D** where, after a 4 h recording in control conditions, 50 nM MC was added and 2 h later, 3 μg/ml LPS was added on top. After 12 h, we washed out the dish for 3 further hours of recording. Although the BD probability plot of **Figure 5D** showed that control data (continuous thick line) were statistically different (*P* < 0.0001) from those obtained in MC (upward triangles), in LPS (inverted triangles) and in washout (thin line), all these last curves were positioned to the left of the control line, and this fact is the opposite of what we reported in **Figures 2C,D** for LPS alone. Moreover, these last three curves were not significantly different from control (*P* > 0.05, using the Kruskal–Wallis test). In **Figures 5E,F** are shown the details of excitability and IBI during an exemplary experiment lasting more than 18 h. The transient depressant effect of MC addition, seen in **Figures 5B,C** is barely visible in excitability, but clearly evident in IBI, which decreased from 45 ± 3.2 s to 26 ± 1.8 s (and SR that increased (excitatory cell in parenthesis) from 1.02 ± 0.15, (*n* = 19) [0.24 ± 0.025,(*n* = 60)] Hz up to 1.38 ± 0.25 Hz, (*n* = 19) [0.30 ± 0.031, (*n* = 60)] not shown. The addition of LPS was followed over ∼10 h, and a weak (∼0.75 and ∼0.5 for inhibitory and excitatory clusters, respectively) *decrease* in excitability and IBI (by a factor of ∼0.5) can be observed before network washout where excitability recovered, but not IBI. Thus, in conclusion the average SR did not significantly change.

Minocycline, at the same concentration, was tested also in some dishes 7–8 h after the LPS application, but no consistent excitability decrease was evident, apart from the fast but transient negative jump already described above in **Figures 5E,F**. We thus conclude that MC at 50 nM was able to effectively inhibit the slow excitability increase produced by LPS, without significantly altering the other properties of the network activity.

### EFFECTS OF CYTOKINE (TNF-α), PURINERGIC ANTAGONIST (PPADS), ANTICONVULSANT CARBAMAZEPINE (CBZ) AND NEUROPROTECTANT THALIDOMIDE

Brain inflammation is characterized by activation of CNS-resident microglia and astrocytes, and it is supposed that these cell types express, release and respond to cytokines (Allan et al., 2005). Indeed, it was suggested in 2002 that glial cells constitutively release the cytokine TNF-α (Beattie et al., 2002) and it was also shown that “synaptic scaling” was dependent on the same cytokine exogenously applied (Stellwagen and Malenka, 2006). On the contrary, it is now clear from transcriptome analysis of purified astrocytes, that this cytokine is only released from microglia (for a review see Béchade et al., 2013). Therefore, we did some tests and verification experiments to determine if our multi-site electrophysiology was able to detect a change in neuronal excitability in response to exogenously applied TNF-α.

Preliminary dose-response experiments suggested that 1 h-long exposures to TNF-α at concentrations in the picomolar range (from 10 to 300 pM, steps: 10, 20, 60, 100, 300), produced a net *decrease* of the inhibitory neuron excitability with no evident effects on excitatory cells as shown in **Figure 6A**. These data, obtained during brief successive dose-steps in a network free of any other drugs, suggest that the cytokine action was relatively fast and the effect of the highest concentration tested was completely washable after a few hours. No changes were present in the down-state duration, i.e., IBI (*n* = 3). On the contrary, in long-term experiments (2 h of control + 15 h in TNF-α + 8 h of recovery), a single application of a small TNF-α concentration (60 pM), produced a delayed and non-washable response characterized by a slow excitability increase in the excitatory neuronal cluster and a decrease in the inhibitory cluster. This is shown in **Figure 6B**, where the excitability data for excitatory and inhibitory neuronal clusters (upper) is shown together with the IBI change (lower). The washout was characterized by a partial 3 h-recovery, and in the further 5 h, the activity during the bursts remained substantially hyperexcitable because the excitatory neurons were more active, probably as a consequence of the lower excitability of inhibitory cells. TNF-α at this low dose, therefore partially mimicked the excitatory action of LPS but this was only apparent on the excitatory neuron clusters and prolonged atypical “seizure-like” burst episodes were not present.

It is now recognized that endogenous adenosine triphosphate (ATP) and ATP (purinergic) receptors play an important role in regulating microglial function, and in particular, the release of cytokines in the brain (Shieh et al., 2014). We therefore performed two experiments designed to investigate the putative role of purinergic receptors in our LPS-induced responses shown above (Pascual et al., 2012). Contrary to our expectation, as shown in **Figure 6C**, pre-conditioning the network with the non-selective P2X receptor antagonist PPADS (50 μM), did not prevent the LPS effects, suggesting that a modulatory effect of endogenous ATP on microglia was not involved.

Next, we were interested to examine the effects of other drugs as putative substitutes of MC as blockers of the LPS effects and tested the effects of the anticonvulsant drug carbamazepine (CBX) and of THAL, also a powerful anticonvulsant (Palencia et al., 2011) and anti-hyperalgesic (Cata et al., 2008). Both agents were tested first for their effects on neuronal excitability. A cumulative CBZ dose-response relation (1, 3, 10, 30, and 100 μM), performed in about 2 h, is shown in **Figure 6D** where a progressively *depressant* action on excitability is clearly evident up to the almost complete silencing of activity at 100 μM. On the contrary, when CBZ or THAL were applied on ultra-long time scales of more than 10 h, as a single dose of 20 and 30 μM, respectively, the actions were *excitatory* but only transient (about 2 h, decaying time constant about 2–3 h, see **Figures 6E,F**) and thereafter the network homeostatically recovered toward its quasi-normal equilibrium level of firing.

After the drug additions, the first effect was a strong and persistent IBI increase (by fourfold for more than 2 h), followed by a delayed hyperexcitability during the bursting activity. These early and late effects may be putatively explained as follows: (1) the initial fast action was probably caused by a generalized hyperpolarization of the resting membrane potential of neurons, with the consequence that fluctuations in their normal “baseline” activity could only rarely attain an amplitude exceeding action potential threshold (leading to the burst onset); (2) the delayed hyperexcitability (more pronounced in inhibitory cells in CBZ) persisted during firing sustained by the putative drug-induced effects on synaptic connectivity and/or ion channel properties. These results led us to conclude that: (1) CBZ concentrations as low as 20 μM (when briefly applied) did not apparently produce remarkable effects, but when applied at steady-state, they caused transient and non-washable long-term effects which were different in the two neuron clusters. In conclusion, the drug cannot be considered useful as a blocker of the very slow LPS effects; (2) likewise for THAL, the data in **Figure 6F** suggest considerations similar to those explained for CBZ: namely, that the drug was acting on time scales and pathways that competed with those activated by LPS, and thus any putative results obtained with it in combination would not clarify the LPS-induced effects in the same manner as shown for MC.

### THE COEXISTENCE OF NEURONS, ASTROCYTES AND MICROGLIA AND THE LPS-INDUCED TNF-α RELEASE IN THE *EX VIVO* LONG-TERM CULTURED NEOCORTICAL NETWORKS

**FIGURE 7:**
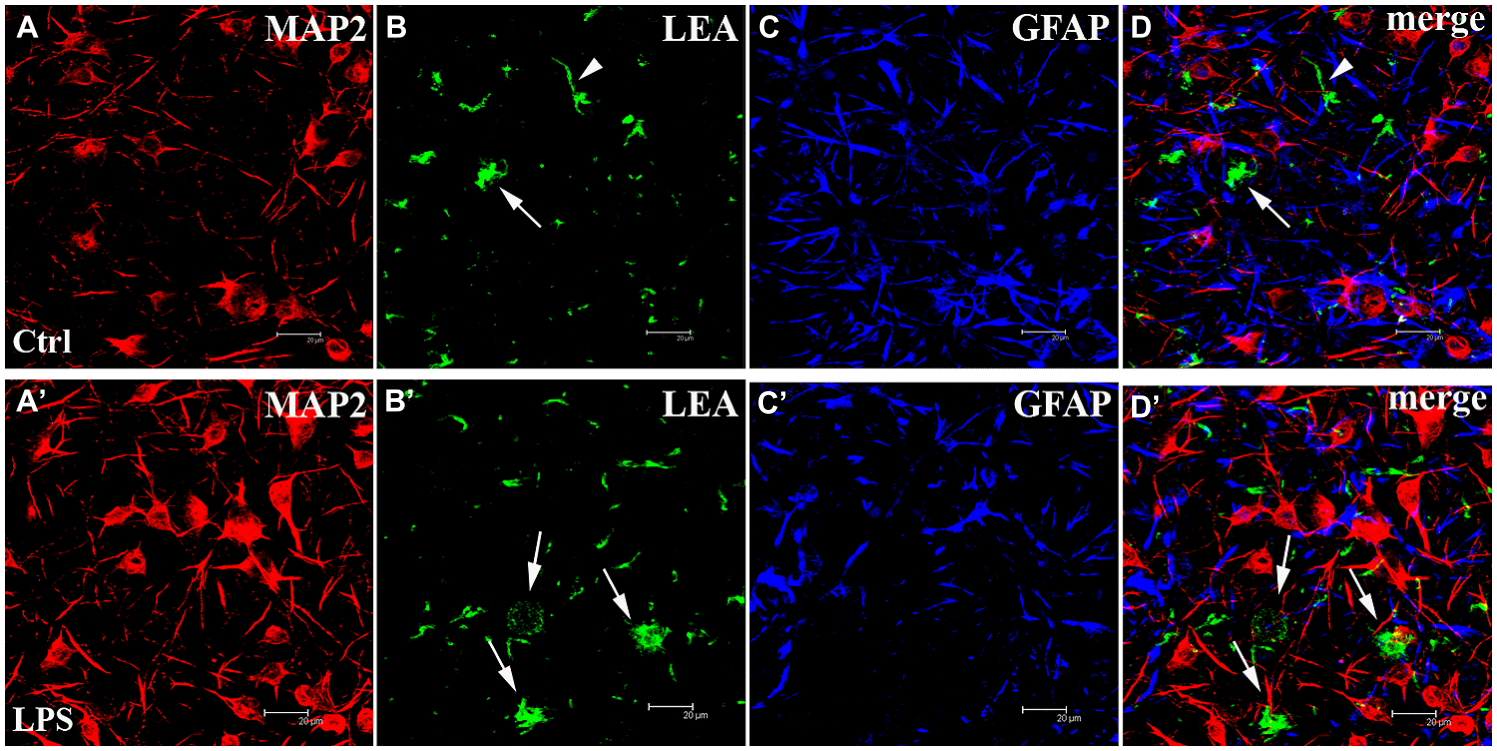
**The cortical networks are a mixture of neurons, astrocytes and microglia.** (Upper panels) Untreated control (Ctrl) culture **(A–D)**. (Lower panels) LPS-treated (LPS) culture **(A’–D’)**. From left to right, confocal microscopical analysis of triple fluorescence staining for: (1) MAP2 (neuronal marker, red signal, **A,A’**), (2) b-LEA lectin cytochemistry (microglial marker, green signal, **B,B’**), (3) GFAP (astroglial marker, blue signal, **C,C’**), (4) **(D,D’)**: merged image of triple labeling. b-LEA lectin-positive (+) cells exhibited both ramified (arrowheads in **B**,**D**, merge) and round amoeboid morphology (arrows in **B,B’,D,D’**) and were sparsely interspersed among MAP2+ neurons and GFAP+ astrocytes. Amoeboid b-LEA lectin+ cells were more frequently observed after LPS treatment (compare **B,B’**) than in controls. Different cell populations in cortical cultures at DIV13. Note that the number of *neurons* in the different pictures was not related to the LPS action. Scale bars: 20 μm.

Finally, two different approaches were used to characterize the cortical cell cultures and to evaluate the microglial population in control and LPS-treated networks: immunofluorescence and tomato lectin cytochemistry methods. The identification of the MAP2-positive neuronal component (**Figures 7A,A**’) showed neurons distributed on the coverslips forming clusters with long dendritic processes. Numerous astroglial cells positive for GFAP (**Figures 7C,C**’) homogeneously surrounded this cortical neuronal network (**Figures 7D,D**’, merge). b-LEA+ microglial cells were scattered in the same fields (**Figures 7B,B**’). In control cultures (i.e., not LPS treated), both ramified resting and amoeboid/activated round microglial cells (Saura, 2007; Kettenmann et al., 2011) were observed (**Figure 7B**). In LPS-treated cultures, b-LEA+ microglial cells appeared more numerous than in control cultures, they showed a predominant round morphology and appeared concentrated in clusters (**Figure 7B**’). The total number of b-LEA+ cells in each examined culture was rather homogeneous both in control and in the LPS group. However, a significant difference was demonstrated by the statistical comparison of the two sets of samples, showing an increase of microglial population after LPS treatment in cell cultures. A quantitative statistical analysis, performed on non-overlapping fields (*n* = 32 for each control and LPS sample, see Materials and Methods), suggests that average microglial cell counts in control (650 cells) and in LPS-treated (852 cells) samples were 18.15 ± 0.98 and 23.75 ± 1.53, respectively (Student’s *t*-test, *P* < 0.01, *n* = 32). Taken together, these results demonstrate that the cells cultured on MEAs include microglia, astrocytes and neurons and that LPS induces a functional increase in the microglial population as expected.

In order to test in our *ex vivo* neocortical networks, the functional state of microglia during the LPS-induced activity and under a MC-pretreatment, we decided to evaluate, by standard ELISA techniques (see Materials and Methods), the release of TNF-α, a cytokine that is universally considered as an index of microglia activation. Nakamura et al. (1999) originally showed that cultured microglia derived from neonatal rats, when stimulated by LPS concentrations as low as 1 μg/ml, release TNF-α with a 1 h lag time; later, interleukins were released and after ∼6 h, NO. In primary retinal microglia cultures, similar effects of LPS were described and in addition, it was shown that MC pretreatment completely blocked the cytokine release (Wang et al., 2005).

**FIGURE 8:**
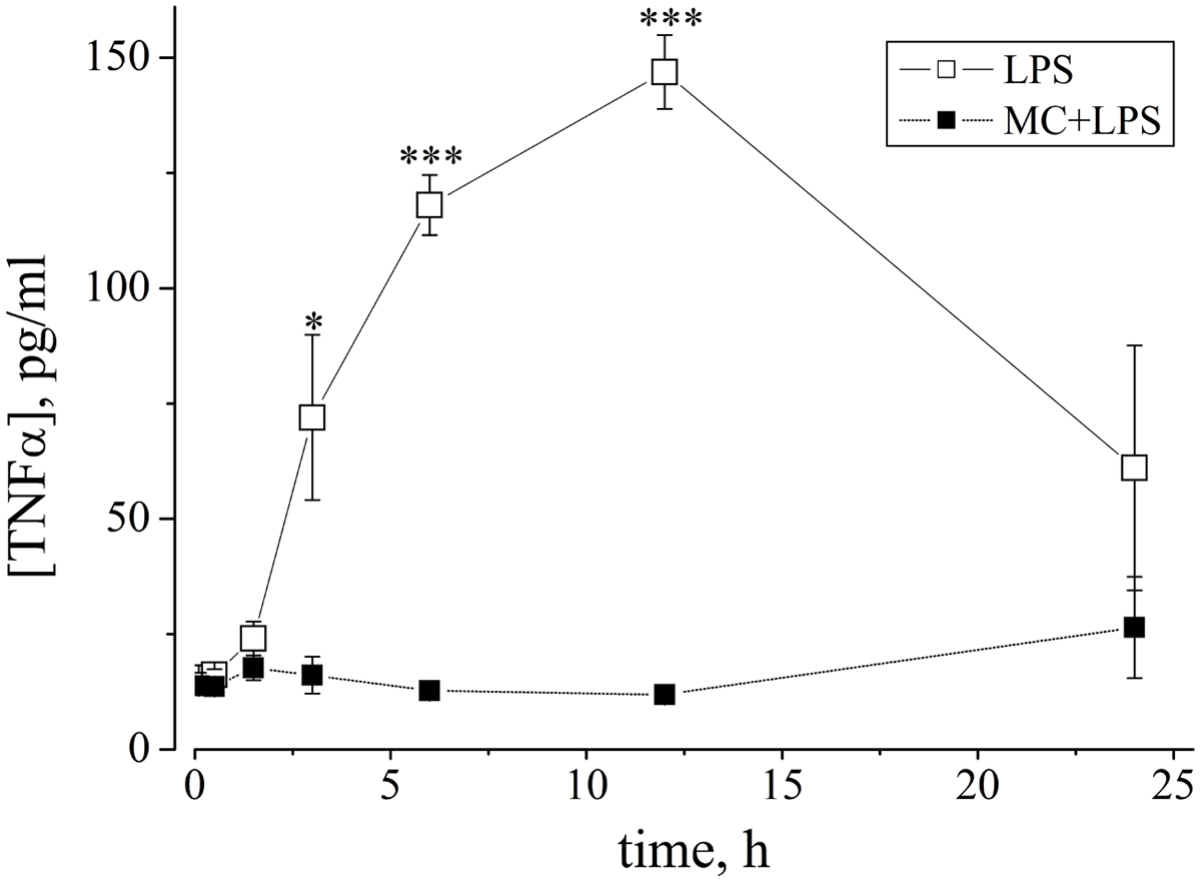
**The cytokine TNF-α is released from LPS-treated networks but not from MC-pre-treated networks.** Graph shows the time-course of changes of TNF-α concentration (ordinate: expressed as pg/ml, see Materials and Methods) vs. time (abscissa: hours), measured from neuronal network culture media, induced by LPS (3 μg/ml) without (open squares) or with (closed squares) MC preconditioning (50 nM, 1 h). Aliquots were collected at different time points (30, 90 min, 3, 6, 12, and 24 h) and assayed with a TNF-α ELISA kit. As shown by asterisks, open square data for LPS alone (mean ± SEM, *n* = 5) were statistically different from data obtained after MC-preconditioning at 3 h with *P < 0.015, and at 6 and 12 h, with ***P < 0.0001.

To gain insight into the time-course of the LPS-induced cytokine release by microglia in our cultures, we collected from culture media, small aliquots before (*t* = 0) and after microglia activation. To perform the measurements, we used a total of 11 MEA dishes; six were treated with LPS alone, and five were pre-treated with MC to check its inhibiting role. The results of our experiments are shown in **Figure 8** where the time-course of the TNF-α concentration is plotted vs. time (open symbols) and with MC pre-treatment (50 nM, 1 h: closed symbols). LPS induced a significant increase in mean TNF-α level at 3, 6, and 12 h (peak) post-LPS treatment, relative to control untreated conditions (where the TNF-α level was very small and at the limit of detection of the method). On the whole, these results indicate that quasi-simultaneously with the prolonged neuronal excitability changes described above, the TNF-α concentration also grew over a similar time-course with respect to control. In 1/6 LPS-experiments, the TNF-α concentration increase was not observed (not shown). Our results thus confirm the functional activation of microglia in our cultures and its LPS-synchronized and MC-sensitive link. It should however be noted, that the peak amplitude of the TNF-α level attained (corresponding to ∼3 pM) is far from that necessary to produce the neuronal effects shown in **Figure 6B**, which were seen at 60 pM of exogenous TNF-α.

## DISCUSSION

### LPS INDUCES LONG-TERM ULTRA-SLOW CHANGES IN NETWORK EXCITABILITY

This study shows that the earlier steps of microglia-induced neuroinflammation induced by LPS can modulate network activity by producing a very slow change of the average firing properties of both excitatory and inhibitory neuronal clusters observed during burst events (without any apparent effect on burst rate), eventually leading to the appearance of abnormally long and atypical “seizure-like” activity in the network. Since the effect was occluded by the anti-inflammatory agent MC at nanomolar concentrations (shown to inhibit activation and proliferation of microglia, see Tikka et al., 2001), we suggest that the hyperexcitability effects are directly mediated by a complement of microglia-released factors (including TNF-α, as we have now demonstrated by ELISA assays; see below) released by LPS in our system, although the exact nature of all these factors and the mechanisms by which they subtly interact to alter cell-firing behavior are currently unclear. Nevertheless, we found that a purinergic antagonist was unable to occlude the LPS-induced effects, supporting a model probably not linked to astrocyte-mediated activity and release of endogenous ATP.

In our exogenous TNF-α-assay experiments in **Figure 6B**, using small non-saturating concentrations of TNF-α (60 pM), we did not reproduce the action of LPS, but instead, we found an un-washable ultra-slow dynamic effect of increasing/decreasing excitability of excitatory/inhibitory neurons, respectively. These data are in agreement with the *in vivo* inhibiting action of exogenous TNF-α injection on kainic acid-induced seizures in mice (Balosso et al., 2005), and with *in vitro* data obtained in rat hippocampal slices, with 100 nM applied TNF-α (Tancredi et al., 1992). The possible mechanisms underlying these TNF-α effects and the role of different MC preconditioning will be further investigated in future experiments.

Clearly, TNF-α *alone*, at the very low pM levels released by LPS in our cultures could not be responsible for the excitability changes we observed. However, it is worth noting that this cytokine has previously been shown to control basal synaptic functions such as plasticity (Stellwagen and Malenka, 2006), attributed to endogenous release by astrocytes, but these cells do not release this cytokine, which is only released by activated microglia (Lehnardt et al., 2002). Stellwagen et al. (2005), also studied the neuronal effects of applying TNF-α in hippocampal cultures and slices but used brief, 15 min, applications at 100 ng/ml (i.e., 2 nM, see the erratum to the Stellwagen et al., 2005 paper appeared in J. Neurosci. Jun 1; 25(22): 1 p following 5454) and demonstrated a differential regulation characterized by synaptic AMPA (α-amino-3-hydroxy-5-methyl-4-isoxazolepropionic acid)-type glutamate receptor (AMPAR) trafficking and GABA_A_R internalization, both by using immunocytochemistry techniques and miniature excitatory/inhibitory synaptic data. Similar applications of TNF-α (but only up to 300 pM), were also performed in our dose-response experiments shown in **Figure 6A** but we observed a dramatic *depression* in excitability of designated inhibitory neuron clusters that was completely reversed in <2 h. These effects resembled those obtained in our laboratory by enhancers of GABAergic neurotransmission such as neurosteroids or benzodiazepines (Puia et al., 2012), though whether a similar mechanism was involved at the level of GABA_A_Rs remains to be determined.

### MICROGLIA AND THE ROLE OF NEUROINFLAMMATION IN CNS DISEASES

Microglia are an integral part of CNS networks, forming the innate defensive system of the CNS and their pathological potential has been extensively investigated (Kettenmann et al., 2011). All CNS diseases involve microglia, which typically convert from the resting/surveillant cell type in the normal brain to an activated form specialized to operate within the diseased environment. In different pathologies, microglia acquire distinct functional states and during the disease progression, modify and change their activated phenotype. Activated microglia specifically interact with neurons and influence their survival either in a positive or in a negative direction. They can physically contact injured neurons and remove synapses, a process termed synaptic stripping (Kettenmann et al., 2013).

Conceptually, microglial cells can affect neural networks either through removal of cellular and subcellular elements (by phagocytosis) or through secreting factors with different properties: transmitter, trophic or neuroprotective. Thus, these factors, released in the activated state, are considered to be pathological signals and, as already mentioned in Introduction, include several types of cytokines, trophic factors, neurotransmitters (ATP and glutamate; Nakajima et al., 2007; Kettenmann et al., 2011; Viviani et al., 2014). On the other hand, studies in cell culture have indicated that microglial cells express a variety of receptors for neurotransmitters, neuropeptides, and neuromodulators and thus have also the capacity to sense neuronal activity (Pocock and Kettenmann, 2007).

Increasing evidence also supports the involvement of inflammatory and immune processes in the etiopathogenesis of seizures. Epilepsy is a disabling neurological disorder that in about 30% of affected individuals is refractory to pharmacological treatment (Perucca et al., 2007). Pharmacological and genetic studies in animal models have shown that specific inflammatory mediators such as cytokines, complement factors and prostaglandins substantially contribute to seizures and that interfering with these molecules or their receptors can reduce seizure frequency and severity (Vezzani et al., 2008).

Although there is substantial evidence that activated microglia can have negative effects in neurologic disease (Bamberger and Landreth, 2002), evidence also exists that under certain circumstances, microglia can be neuroprotective. Several groups have found that microglia express the glutamate uptake transporter, which may help microglia protect nerve cells during excitotoxic injury (Schwartz et al., 2003).

### MECHANISM OF LPS ACTION

Small amounts of LPS from invading bacteria are one of the first signals detected by the body upon infection, and detection of LPS primes the immune system to mount a defense. Under some circumstances, the initial inflammatory response can become uncontrolled and ultimately lead to other deleterious effects including prolonged inflammation and cytokine release which is known to contribute to CNS dysfunction, chronic depressive disorders and neurodegenerative processes (Perry, 2004; Kettenmann, 2007). Although the CNS actions of pro-inflammatory cytokines have been implicated in “sickness behavior” that develops during the course of an infection (Cunningham et al., 2002), the mechanisms in the brain that trigger this adaptive behavioral response are not well understood. The Toll-like receptor 4 (TLR4) and its potent ligand LPS, represent one of the first and best characterized receptor/ligand systems (Gertig and Hanish, 2014). TLR4 receptors are expressed on microglia and not astrocytes (Lehnardt et al., 2002).

Systemic LPS acts on the CNS through several parallel pathways (Konsman et al., 2002) and given that TLR4 receptors are expressed in the brain (Chakravarty and Herkenham, 2005) and high LPS concentrations (100 μg/ml) lower the seizure threshold in rodents (Sayyah et al., 2003; Galic et al., 2008), its pathways have been investigated in some detail. Indeed, increasing evidence indicates that, in the absence of pathogens, TLR signaling can be activated also by molecules released by injured tissue (Bianchi and Manfredi, 2009) and among these is the high-mobility group box 1 protein (HMGB1), released by neurons and glia, that binds to TLR4 receptors (Scaffidi et al., 2002; Park et al., 2004; Maroso et al., 2010). Preliminary experiments done in our laboratory with increasing concentrations of HMGB1, produced slow actions similar to those reported here for LPS (Gullo et al., in preparation). Very recently, HMGB1 pulses have also been reported to enhance focal seizure generation in a brain slice preparation (Chiavegato et al., 2014). The exact mechanism by which LPS activation of TLR4 signaling in our neuron system led to slow changes in excitability remain to be determined, although it is interesting to note that in a study of LPS action on dorsal root ganglion neurons recorded *in vitro*, a clear increase in cell excitability was observed, possibly through subtle changes in the density and gating of voltage-gated Na^+^ channels involved in neuronal firing (Due et al., 2012). Moreover, also in acute hippocampal slices, 10 μg/ml LPS facilitated epileptiform activity (induced by Mg^2+^-free ACSF + 4-AP) via enhanced excitatory synaptic transmission and release of TNF-α and IL-1β (Gao et al., 2014).

### PREVIOUS LPS-INDUCED *IN VITRO* STUDIES IN BRAIN SLICES, ORGANOTYPIC AND DISSOCIATED CELL CULTURES

Depending on the LPS concentration used, the effects observed on single neurons or in brain slice activity can be either transient or sustained and can initiate irreversible changes like apoptosis. Moreover, the LPS-induced release of cytokines, chemokines and NO have been generally studied over several hours (Nakamura et al., 1999; Wang et al., 2005). The effects of LPS-induced microglial secretion of TNF-α and the consequent modulation of neurotransmission has also been studied in acute brain slices and in cultured cells as recently reported (Nimmervoll et al., 2012; Pascual et al., 2012). In H. J. Luhmann’s laboratory, the experiments were both performed in organotypic and primary dissociated cells cultures by using an LPS concentration which caused rapid changes in spontaneous synaptic activity and caspase-3-dependent cell death in neurons, with no effects on astrocytes (Nimmervoll et al., 2012). Indeed, the LPS concentration used by these authors was more than three times higher (10 μg/ml) than our concentration and interestingly, their laboratory used the same multisite MEA recording dishes as ours. Unfortunately, in both brain slices and cultured neuron dishes, no spike sorting to find different units was performed. With respect to control, their results 1 h after the LPS treatment resulted in a significantly higher IBI between bursts (“oscillations”) for slices and a “desynchronization” of the networks in dissociated neuronal cultures. On the whole, their results suggest that LPS concentrations of 10 μg/ml (higher than ours of 3 μg/ml) were able to produce after 2 h, large increases in TNF-α and macrophage inflammatory protein 2, but induced unwanted “toxic” effects, difficult to be compared with ours that were always recoverable on washout. Similar detrimental changes may also have occurred after chronic (7 day) exposure of organotypic hippocampal slices to LPS, which led to a persistent *decrease* in intrinsic neuronal excitability (Hellstrom et al., 2005).

Pascual et al. (2012) essentially carried out the same experiment in acute hippocampal brain slices as we did in dissociated neuron networks (with different techniques), for studying the LPS-induced excitability, by preconditioning networks with MC, and found exactly the same occlusion effect. Interestingly, they checked the TLR4 role in these experiments by also using TLR4 -/- KO mice and concluded that microglia are required for LPS to modulate neuronal activity. However, they observed only a transient (few minutes, but repeatable response) effect of LPS and ultra-slow effects were not studied as we did here. On the contrary, although we can exclude an important role for purinergic receptors during our experiments (see **Figure 6C**), they suggested that these receptors are “necessary” for the positive modulation of the synaptic mEPSC frequency (same purinergic receptor blocker at the same concentration). It is not easy to compare our and their experiments both on an electrophysiological and biological basis: we measured excitability of neurons and not mEPSCs, and in conclusion, we tested the functional outcome of the global synaptic bombardment in both neuronal clusters (simultaneously in many neurons), while they tested the miniature excitatory input and not the functional outcome, i.e., spikes in various principal neurons, not simultaneously. Interestingly, they also described an LPS-induced seizure-like activity, but only under facilitated conditions (0 external Mg^2+^ medium + block of GABA_A_Rs with picrotoxin). In our experiments, LPS induced burst-like epileptiform events without background pharmacological intervention (see also Gao et al., 2014), most likely through the induced slow changes in overall network excitability.

On the whole, these data strongly suggest that the LPS-induced effects, seen in other laboratories in cell preparations containing viable microglia (releasing TNF-α) identified astrocytes and spiking neurons, have relatively large differences within themselves and with respect to our data. As reported, LPS concentrations at 1 μg/ml or below, strongly reduced successful experiments and statistical significance. On the contrary, the advantage of our *ex vivo* networks as compared to the other *in vitro* preparations, was the possibility to record: (1) stable excitable activity for hours in control, (2) follow long-term changes induced in LPS, and (3) during the action of picomolar TNF-α (as shown in **Figure 6A**); moreover, at the same time, we were able to test the effects of other drugs selective for different pathways, as shown in **Figure 6**).

### EFFECTS OF MINOCYCLINE

Minocycline is a semi-synthetic, second-generation tetracycline antibiotic analog which effectively crosses the blood–brain barrier. It was first reported that MC had neuroprotective effects in animal models of ischemic injury (Yrjanheikki et al., 1998). It has been shown that this action of MC involves not only microglia but also T cells and their subsequent microglial activation (Giuliani et al., 2005); moreover, it attenuates the production of TNF-α in neuron/glia co-cultures (Lee et al., 2004). More recently, it was reported that MC promotes re-myelination in rat brain cultures (Defaux et al., 2011). These results suggest that MC attenuates microglial reactivity and favors re-myelination by enhancing the differentiation of oligodendrocytes. There are reports where MC has consistently been used in the 40–100 μM range, which is three orders of magnitude larger than the concentration used in our experiments (60 nM). We used the MC concentration reported by Tikka et al. (2001), which is one of the lowest reported in the literature, although in some papers, concentrations up to 60 μM were used in LPS-induced retinal microglia activation (Wang et al., 2005). MC was also tested for its effects on Na^+^, K^+^, and Ca^2+^ ion channels in hippocampal neurons, where it produced only weak inhibitory effects, but it has been suggested to reduce the release of glutamate with an EC_50_ of 60 μM.

Minocycline is an unusual type of tetracycline because its use is still increasing and has attracted many research areas of application, but it is no longer considered an antibiotic (see Garrido-Mesa et al., 2013). In addition to its own antimicrobacterial properties, MC has been reported to exert neuroprotective effects in various experimental models such as cerebral ischemia, traumatic brain injury, amyotrophic lateral sclerosis, Parkinson’s disease, kainic acid treatment, Huntington’ disease, and multiple sclerosis. Recently, the effect of MC in Alzheimer’s disease has been also reported in mouse models at a concentration of 10 mg/kg/day and apparently it is not dangerous, but when given in Aβ-treated mice, the MC concentration that was necessary to improve latency times in behavioral tests had to be increased by five times (Choi et al., 2007). We also performed long-term experiments in our networks with MC at 2 μM and after a sudden 50% decrease of excitability, IBI slowly increased by the same amount producing a net marked SR decrease in the following 8 h, thus confirming that the network was substantially unrecoverable (not shown).

## CONCLUSION

In conclusion, our study provides evidence that it is possible to characterize the network excitability and its response to the pro-inflammatory agent LPS in a standard culture of dissociated cortical neurons, astrocytes and microglia grown on a MEA recording environment. Our results show that this response can be prevented by pre-exposure of the network to a low dose of the anti-inflammatory drug MC. In view of the known involvement of neuroinflammatory components in a wide range of neurodegenerative, neurological, as well as psychiatric central nervous disorders, we would like to suggest that the use of anti-inflammatory agents like MC might be useful as adjuncts to conventional therapies in the management of these debilitating conditions. The fact that we can detect and analyze long-term effects on neuron activity during microglia activation also opens the possibility to study recently demonstrated roles of “resting” microglia, probably bi-directionally cross-talking with neurons in the developing CNS (Béchade et al., 2013; Kyungmin et al., 2013; Schafer et al., 2013; Sheridan and Murphy, 2013).

## AUTHOR CONTRIBUTIONS

Enzo Wanke conceived and designed the research project. Francesca Gullo did all the MEA experiments on the dissociated cultures and performed the preliminary analysis of the data. Alida Amadeo did the immunohistochemistry analysis. Barbara Costa designed the TNF-α release experiments and Giulia Donvito performed the experiments. Marzia Lecchi, Andrew Constanti and Enzo Wanke wrote the manuscript.

## Conflict of Interest Statement

The authors declare that the research was conducted in the absence of any commercial or financial relationships that could be construed as a potential conflict of interest.
